# An update on radiomics techniques in primary liver cancers

**DOI:** 10.1186/s13027-022-00422-6

**Published:** 2022-03-04

**Authors:** Vincenza Granata, Roberta Fusco, Sergio Venazio Setola, Igino Simonetti, Diletta Cozzi, Giulia Grazzini, Francesca Grassi, Andrea Belli, Vittorio Miele, Francesco Izzo, Antonella Petrillo

**Affiliations:** 1 Division of Radiology, “Istituto Nazionale Tumori IRCCS Fondazione Pascale – IRCCS di Napoli”, Via Mariano Semmola 80131, Naples, Italy; 2 Medical Oncology Division, Igea SpA, Napoli, Italy; 3 Department of Radiology, Azienda Ospedaliero-Universitaria Careggi, Florence, Italy; 4 Italian Society of Medical and Interventional Radiology (SIRM), SIRM Foundation, Via Della Signora 2, 20122 Milan, Italy; 5 Division of Radiology, “Università Degli Studi Della Campania Luigi Vanvitelli”, Naples, Italy; 6 Division of Hepatobiliary Surgical Oncology, “Istituto Nazionale Tumori IRCCS Fondazione Pascale – IRCCS di Napoli”, 80131 Naples, Italy

**Keywords:** Radiomics, Texture analysis, Machine learnings, Hepatocellular carcinoma, Cholangiocarcinoma

## Abstract

**Background:**

Radiomics is a progressing field of research that deals with the extraction of quantitative metrics from medical images. Radiomic features detention indirectly tissue features such as heterogeneity and shape and can, alone or in combination with demographic, histological, genomic, or proteomic data, be used for decision support system in clinical setting.

**Methods:**

This article is a narrative review on Radiomics in Primary Liver Cancers. Particularly, limitations and future perspectives are discussed.

**Results:**

In oncology, assessment of tissue heterogeneity is of particular interest: genomic analysis have demonstrated that the degree of tumour heterogeneity is a prognostic determinant of survival and an obstacle to cancer control. Therefore, that Radiomics could support cancer detection, diagnosis, evaluation of prognosis and response to treatment, so as could supervise disease status in hepatocellular carcinoma (HCC) and Intrahepatic Cholangiocarcinoma (ICC) patients. Radiomic analysis is a convenient radiological image analysis technique used to support clinical decisions as it is able to provide prognostic and / or predictive biomarkers that allow a fast, objective and repeatable tool for disease monitoring.

**Conclusions:**

Although several studies have shown that this analysis is very promising, there is little standardization and generalization of the results, which limits the translation of this method into the clinical context. The limitations are mainly related to the evaluation of data quality, repeatability, reproducibility, overfitting of the model.

*Trial registration*: Not applicable.

## Introduction

Radiomics is an emerging field that extracts and analyses data from medical images, comprising quantitative and qualitative features not detected by human eye [1–6]. The Radiomics analysis includes several moments: image acquisition (all radiological or nuclear medicine procedures are involved); the segmentation of volume of interest by automatic, semi-automatic or manual segmentation tools; features creation; database improvement; database analysis with the construction of a predictive model and the validation of the models created and Radiomics signature [7–15].

The features can be morphological, of First-, Second- and Higher-ordes. Morphological ones describe the shape of the traced region of interest (ROI) and its geometric properties such as volume, maximum diameter along different orthogonal directions, maximum surface, tumour compactness, and sphericity. First-order statistics features describe the distribution of individual voxel values without concern for spatial relationships.

These are properties based on the histogram that report the average, median, maximum and minimum values of the intensity of the voxels, their asymmetry (asymmetry), kurtosis (flatness), uniformity and entropy. Second-order statistical characteristics include structural characteristics, which are obtained by calculating statistical interrelationships between neighboring voxels and provide a measure of the spatial arrangement of the voxel intensities. Higher-order statistical characteristics are obtained with statistical methods after applying filters or mathematical transformations to images such as fractal analysis, Minkowski functional, wavelet transforms and Laplacian transforms of Gaussian filtered images [16–22].

Clinical, pathological and genomic relationships are established for the predictive model. Therefore, this analysis allows the integration of radiomic characteristics and molecular, clinical or other data of the patient allowing to obtain precision medical instruments [20–23].

Today, the main relevance area is oncological setting, since Radiomics features, providing data on tumour or tissue microenvironment, could be associated with histological grade, prognosis, response to therapy, and survival in innumerable cancers [22, 24–31]. The possibility to combine radiomics with genomic data (“radio-genomics”) could theoretically offer the highest level of personalized risk patients stratification so that to greatly augment patient selection for different cancer therapy [32–40].

With the development of the deep learning (DL) technique, the neural network is more commonly used in radiomics studies, and has achieved expert-level performance in several tumours [41–45]. DL self-learning quantitative features may supplement unrevealed imaging features besides conventional radiomic features to improve the predictive power. Additionally, DL-based radiomics avoided time-consuming [45, 46].

Recently, it has been a significant increase in the radiomics investigation in liver disease, including liver fibrosis assessment, characterization of malignant and benign lesions, and prognosis [47–49].

## Methods

This article is a narrative review on Radiomics in Primary Liver Cancers. Particularly, limitations and future perspectives are discussed.

## Results

### Radiomics: Basic principles and process

Radiomic is planned to be utilised in precision medicine decision support, employing standard of care images that are usually obtained in clinical setting [50–53]. Moreover, Radiomics offers prognostic biomarker which allow for a fast, low-cost, and repeatable means for longitudinal analysis [54–56]. Radiomics is based on the features extraction from medical images, [57–60]. Images assessed during this analysis are accumulated from different centers or data-centers; so, these images could be obtained employing different manufacturers, with diverse protocols and parameters. These elements could influence radiomic models [61, 62].

Segmentation phase is a crucial moment since features are obtained from the segmented volumes. This step is difficult since several lesions have unclear borders and could soffer of high inter-reader variability. However, several researches believe that this approach by expert operator should be chosen even if it is time consuming and not always feasible due to very large data sets to analyse [63]. Automatic and semi-automatic approaches have been developed. Common requirements include maximum automaticity with minimum operator interaction, time efficiency, accuracy, and boundary reproducibility. Several algorithms rely on region-growing methods that require an operator to select a seed point within the volume of interest [63].

Generation of features refers to the extraction of Semantic Features such as dimension, necrosis, margin, location or extraction of non semantic features such as shape, histogram or texture [63–69].

Texture analysis (TA) has sparked interest in the clinical setting as it has proven to be a substantial computer-assisted diagnostic tool [70–72]. TA could be defined as the spatial arrangement of models that provides the visual aspect of coarseness, randomness, smoothness. The use of TA compromises the classification and segmentation of the target area, involving a six-step method: image acquisition, definition of the region of interest (ROI), ROI pre-processing, feature extraction, feature selection and classification [70–72]. Manual ROI delineation is still considered the preferred approach. The size of the ROI should be large enough to get the plot data thereby producing statistical significance. It has been established that some characteristics are related such as texture and ROI characteristics, such as mean intensity and variance [73–77].

Feature extraction is the main step in TA indicating the computation of texture features from predefined ROIs. Several methods have been offered, including 2D methods or 3D approaches. Application of 4D TA is promising by including the temporal dimension available in some MRI datasets [78–81].

Radiomic analysis should be performed with large data sets to obtain high statistical power [82–87]. We often have datasets with a large number of features extracted from images with a low number of cases. Often many of the characteristics extracted are redundant, non-informative and not useful in the database. Therefore, initial analyzes should include dimensionality reduction and feature selection, generally achieved through unsupervised approaches; and association analysis with one or more specific outcomes using supervised approaches. The two most commonly used unsupervised methods are cluster analysis and principal component analysis (PCA). All selected features that are considered reproducible, informative, and non-redundant can then be used for association analysis. Supervised multivariate analysis consists of building a mathematical model to predict a result or a response variable. The different analysis approaches depend on the purpose of the study and the category of results ranging from statistical methods to data mining / machine learning approaches, such as random forests, neural networks, linear regression, logistic regression, absolute minimum shrinkage and selection operator, and Cox proportional hazards regression [88–92]. Furthermore, since models must be validated to be preferably run on outpatient and independent patient groups, comparability of features extracted from images with different parameters and segmented with different techniques is challenging and can affect the final performance of the model itself [92].

**Fig. 1:**
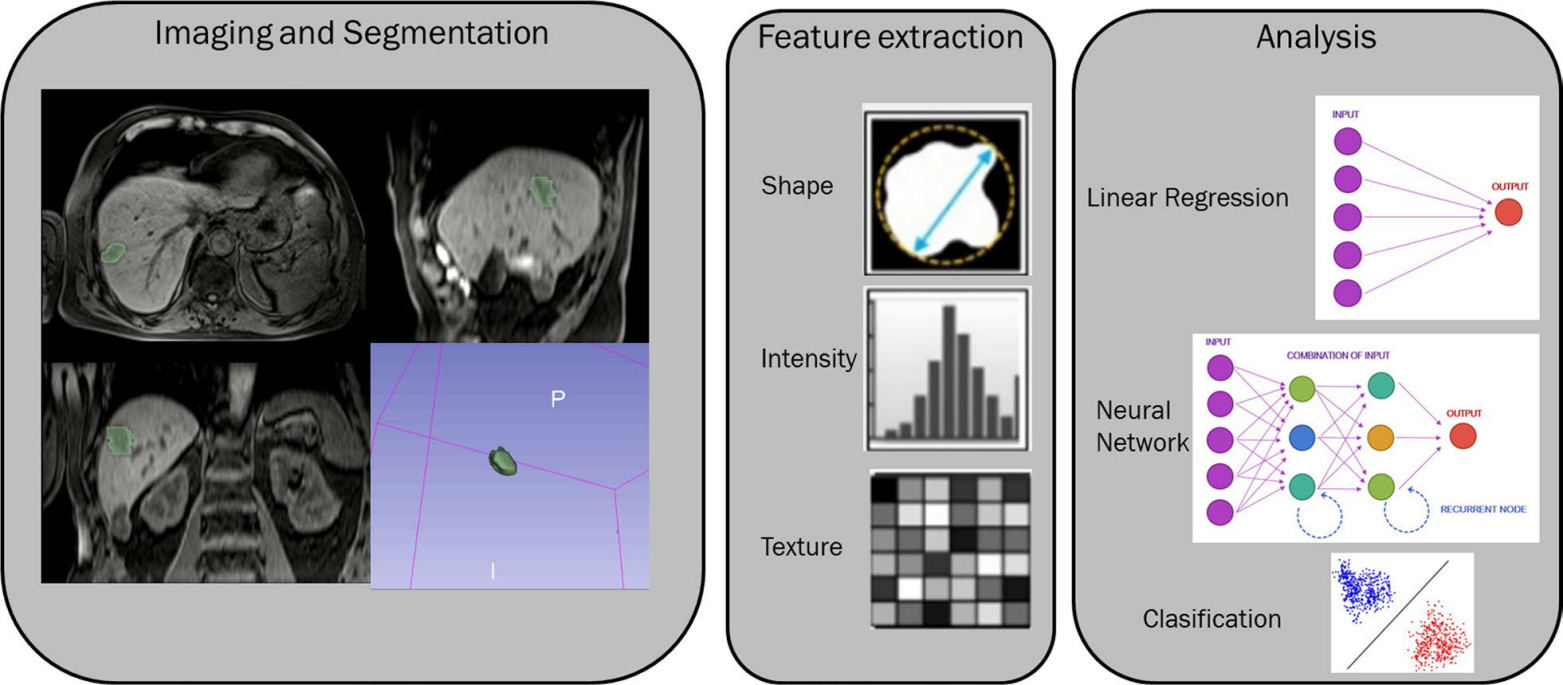
Workflow for extraction of radiomic features from clinical images. The first step contains receiving images and segmentation of the region of interest. The next step is feature extraction within the defined ROI. In the last step the features are analysed, and combined and compared with clinical data

Figure 1 reports a graphic representation of the workflow for extraction of radiomic features from clinical images.

### Current applications

#### Clinical setting

Primary liver cancer, including hepatocellular carcinoma (HCC), intrahepatic cholangiocarcinoma (ICC) and other rare types, is the sixth most usually detected tumour and the third leading cause of tumour death worldwide. HCC may be noninvasively diagnosed by imaging findings alone, often without biopsy. Several morphological and functional data obtained during imaging studies allow a truthful ICC diagnosis.

The choice of modality (CT, US/CEUS or MRI) is correlated to patient, department, and regional features [50, 51, 51, 93–106].

**Fig. 2:**
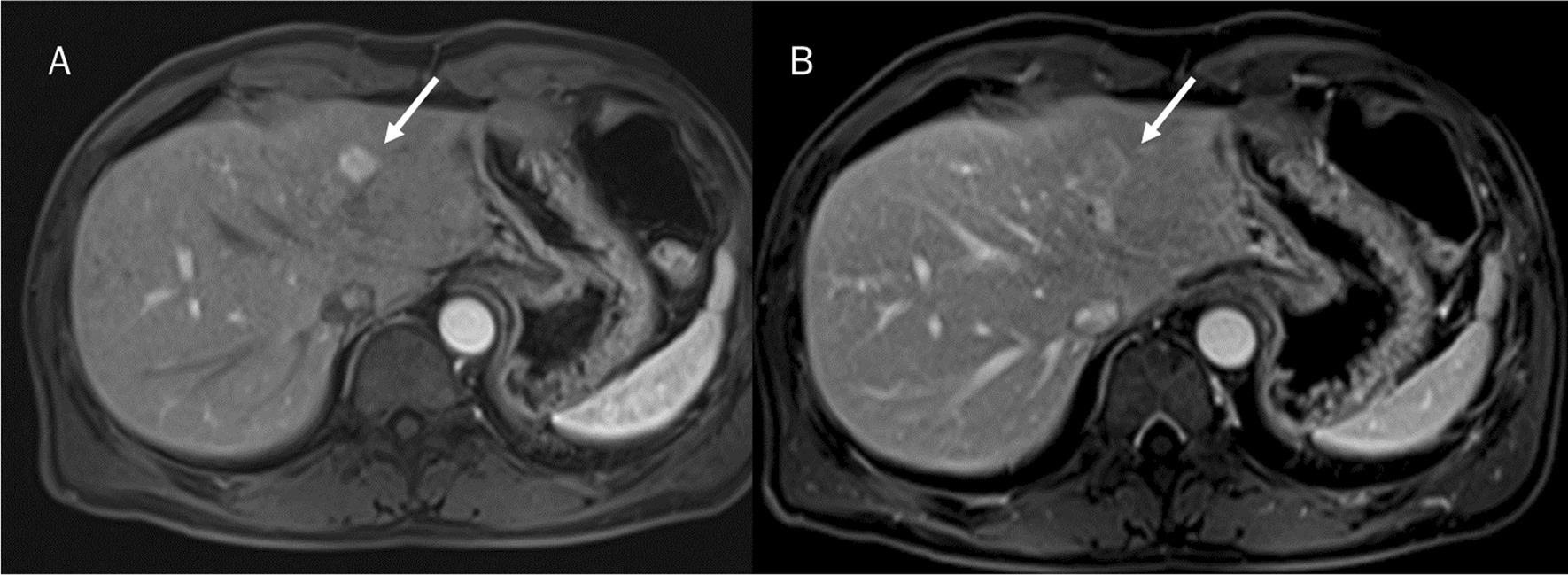
HCC on II liver segment assessed by MRI. In **A**, arterial phase, the arrow shows lesion arterial hyperenhancement. In **B**, portal phase of contrast study, the arrow shows lesion wash-out and capsule appearance

**Fig. 3:**
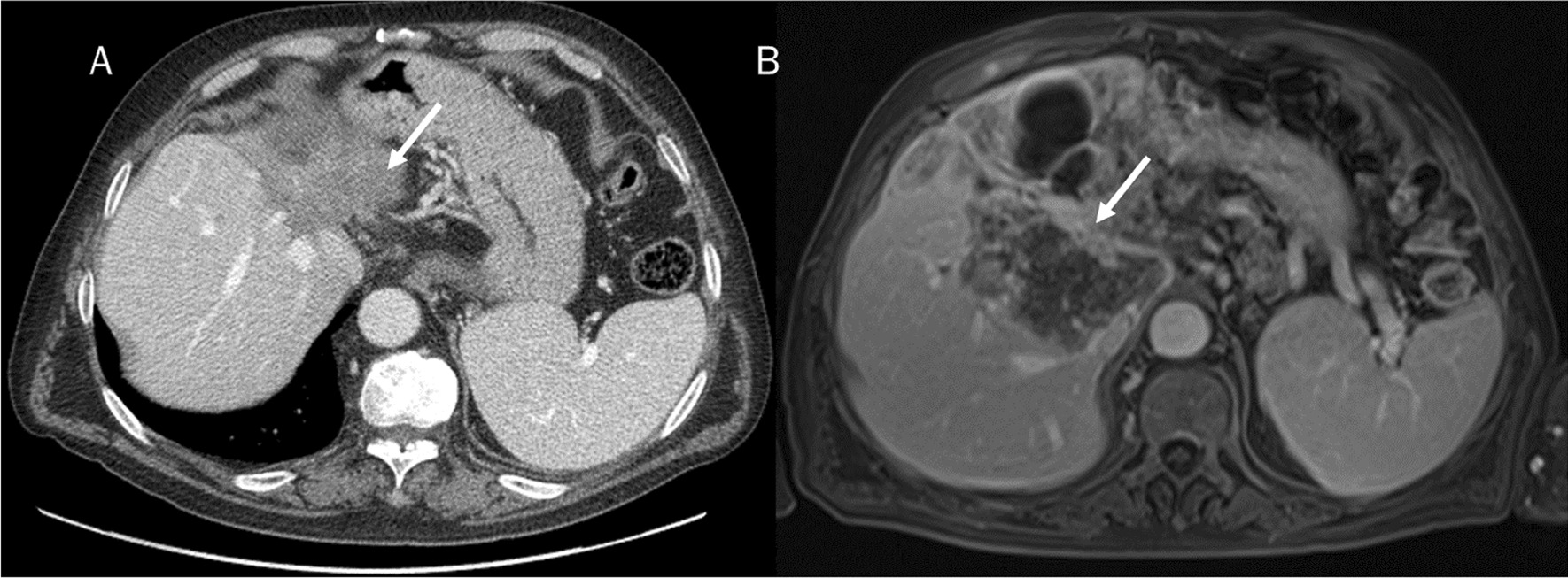
ICC on II-IV hepatic segment assessed by CT (**A**) and MRI (**B**). The lesion shows peripheral rim hyperenhancement with progressive contrast enhancement (arrow)

Radiomics is a promising tool in the assessment of HCC (Fig. 2) and ICC patients (Fig. 3).

#### HCC and radiomics

Up to now, several researches have assessed radiomics or radiogenomics features as biomarkers in HCC patients, evaluating correlation with response and/or recurrence after several treatments as chemotherapy, resection, ablation therapies, and transplantation. The main diagnostic tool used in radiomics studies has been Computed Tomography (CT) [107]. Otherwise, few researches have assessed radiomics data obtained by Magnetic Resonance Imaging (MRI). This may be due to the difficulty of providing standardization of MR sequences respect to CT [23, 108, 109]. In fact, MRI offers the advantage of high contrast resolution and functional data, and this tool is superior in the assessment of tumour metabolism.

The areas of main attention have been correlated to lesion characterization and treatment evaluation [107, 110–135].

For differentiation between HCC and benign liver lesions, a CT radiomic nomogram demonstrated an area under the receiver operating characteristic curve (AUC) of 0.917 to differentiate focal nodular hyperplasia (FNH) by HCC [129]. An MRI reported an AUC of 0.89 (sensitivity 0.822, specificity 0.714) to differentiate HCC by haemangioma using conventional and diffusion sequences (DWI) [130]. Furthermore, using CT and MRI, fusion models showed an AUC of 0.966 for CT and 0.971 for MRI to differentiate HCC by FNH [131]. Furthermore, a retrospective multicenter cohort study of 178 patients with cirrhosis showed an AUC of 0.66 in the diagnosis of HCC, demonstrating the advantage of Artificial Intelligence (AI) to improve physician decisions identifying high risk of HCC patients [188].

Oyama et al. evaluated MRI radiomics features for liver tumor classification using TA and topological data analysis demonstrating that TA could provide useful data for diagnosing liver disease [136]. Li et al. assessed the TA feasibility based on the recovery of attenuated spectral inversion on MRI for the classification of hepatic haemangioma (HH), liver metastases (HM), and hepatocellular carcinoma (HCC), demonstrating that HH versus HM, HM versus HCC and HH and HCC could be differentiated by 9, 16 and 10 TA features, respectively [137]. Jansen et al. assessed MRI datasets of 125 benign lesions (40 adenomas, 29 cysts and 56 haemangiomas) and 88 malignant lesions (30 HCC and 58 metastases). Contrast curve, grey level histogram, and grey level co-occurrence matrix texture features were extracted from the DCE-MRI and T2- W images. In addition, risk factors including the presence of steatosis, cirrhosis, and a known primary tumour were used as features. Fifty features with the highest ANOVA F- score were selected and fed to a randomized trees classifier. The classifier evaluation was performed using the leave-one-out cross validation and ROC curve analysis was performed. They demonstrated that the overall accuracy for the classification of the five main types of focal liver disease is 0.77. Sensitivity / specificity is 0.80/0.78, 0.93/0.93, 0.84/0.82, 0.73/0.56 and 0.62/0.77 for adenoma, cyst, haemangioma, hepatocellular carcinoma and metastases [138], respectively. These data are similar to the data by Gatos et al. [139].

Different researches have suggested the use of radiomic parameters to guide therapeutic decisions by response prediction of ablative therapies and immuno-oncological characteristics [124–126]. Therefore, if the therapeutic direction is considered inappropriate for ablation treatment, it should be changed with the targeted molecular agents use. However, the clinical benefit of radiomic features should been validated by further studies in a prospective setting.

#### ICC and radiomics

Many studies have been assessed the radiomics or radiogenomics role in ICC [140–166]. The main interest area has been the assessment of recurrence after surgical resection.

Chu et al. [140] assessed 203 ICCs, that were subdivided into training and the validation set. Clinical features and radiomic features were used with a random forest algorithm and logistic models to build both a clinical model and a radiomic model. The radiomic model showed a higher AUC than the clinical model to predict avoidable resections in ICC reaching a sensitivity of 0.846 and a specificity of 0.771 in the validation cohort [140]. Quin et al. [141] developed a multilevel model, integrating clinicopathology, molecular pathology and radiology to predict early recurrence after curative surgery, using a machine-learning analysis of 18,120 radiomic features based on CT studies and 48 clinical features. They demonstrated that the radiomics based multilevel model has superior performance over conventional staging systems and could serve as a prognostic tool for planning surveillance and guiding individualized post-operative management [141]. Also Hao et al. [142] developed a non-invasive CT based radiomics analysis model to predict early recurrence in 177 ICC patients. Radiomic features were extracted on six established radiomic models were selected as stable according to the robustness-based rule. Max-Relevance Min-Redundancy (MRMR) combined with Gradient Boosting Machine (GBM) produced the highest AUCs of 0.802 and 0.781 in the training and testing set, respectively [142].

Mosconi et al. [150] evaluated the relationships between the structural features of CT before TARE and objective response (OR), progression-free survival (PFS) and overall survival (OS). They demonstrated that iCCs showing OR after TARE had higher contrast iodine uptake in the arterial phase (higher mean histogram values, *p* < 0.001) and a more homogeneous distribution (lower kurtosis, *p* = 0.043; GLCM contrast, *p* = 0.004; GLCM dissimilarity, *p* = 0.005 and higher GLCM homogeneity, *p* = 0.005 and GLCM correlation *p* = 0.030) on pre-TARE CT. A favourable radiomic signature was calculated and observed in 15 of the 55 patients [150].

#### Current limitations

To day, radiomics researches are in their immaturity with no standardized or unified standards for this problematic analysis. Though several researches reported meticulously manual segmentation, however, automated segmentation algorithms should be employed for realising standardization. In addition, the absence of clear definition of reason for false-positive results in Convolutional Neural Network still an important issue. In addition, for ROI selection, there is no appropriate algorithm to segment tumour area. Another critical issue is the lack of standardization in results reporting that makes it confusing for readers. It may be appropriate report features according to ‘Image biomarker standardisation initiative’ using formal lexicon.

With regard to machine-learning algorithms, these are different in different studies since there is no research to prove which algorithm is the main suitable considering the study type. Finally, most of the current research results are still in the training sample stage, so the model's high accuracy does not reflect its actual predictive ability. Whether the model is really effective or not depends on the validation phase by the test sample.

## Discussion and Conclusions

Radiomics is a rapidly evolving field of research that deals with the extraction of quantitative metrics within medical images that capture tissue and lesion characteristics such as heterogeneity and shape and which can, alone or in combination with demographic, histological, genomic or proteomic data, to be used for the resolution of clinical problems. In oncology, the assessment of tissue heterogeneity is of particular interest: genomic analyzes have shown that the degree of tumour heterogeneity is a prognostic determinant of survival.

Although many studies have shown that radiomics to be very promising, there has been little standardization and generalization of radiomic findings, which limit the use of this method into the clinical practice. Clear limitations especially regard to data quality control, repeatability, reproducibility, generalizability of results and issues related to model overfitting.

## Data Availability

Data are available at https://zenodo.org/record/6307725#.Yhz8VOjMK3A.
